# Thin Films of α-Quartz GeO_2_ on TiO_2_-Buffered Quartz Substrates

**DOI:** 10.1021/acs.cgd.3c00476

**Published:** 2023-12-18

**Authors:** Silang Zhou, Kit de Hond, Jordi Antoja-Lleonart, Václav Ocelík, Gertjan Koster, Guus Rijnders, Beatriz Noheda

**Affiliations:** † Zernike Institute for Advanced Materials, University of Groningen, Nijenborgh 4, 9747 AG Gronigen, The Netherlands; ‡ MESA+ Institute for Nanotechnology, University of Twente, P.O. Box 217, 7522 NH Enschede, The Netherlands

## Abstract

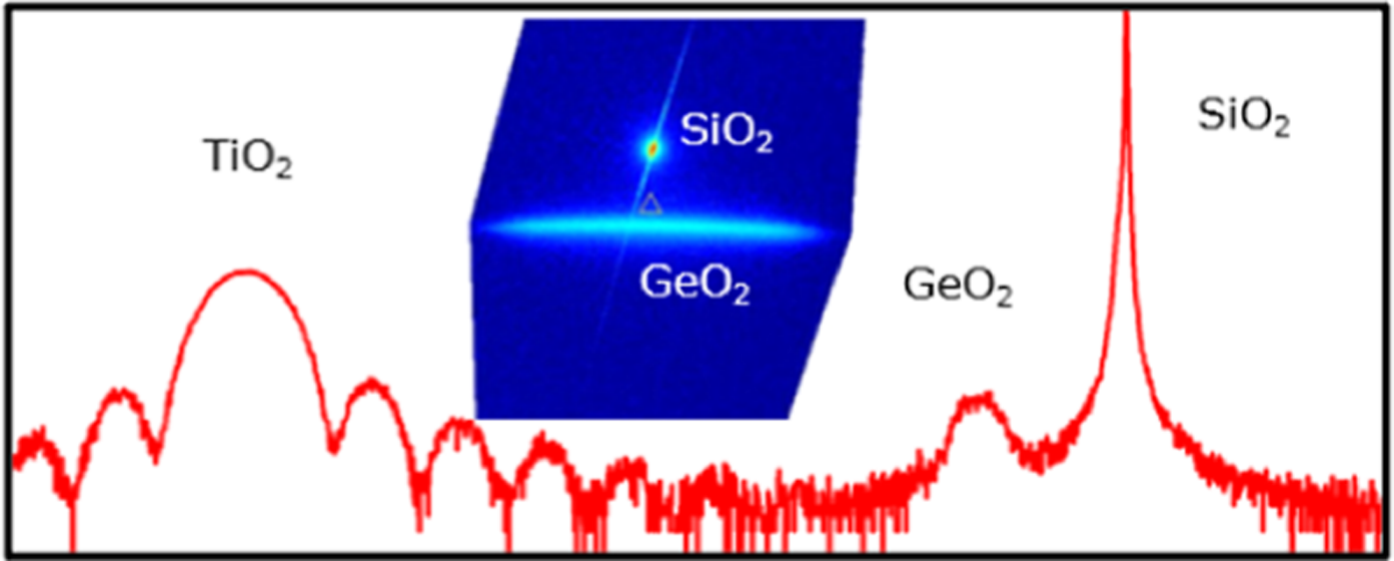

α-Quartz (SiO_2_) is one of the most widely used piezoelectric materials. However, the challenges associated with the control of the crystallization and the growth process limit its production to the hydrothermal growth of bulk crystals. GeO_2_ can also crystallize into the α-quartz phase, with a higher piezoelectric response and better thermal stability than SiO_2_. In a previous study, we have found that GeO_2_ crystallization on nonquartz substrates shows a tendency to form spherulites with a randomized orientation; while epitaxial growth of crystalline GeO_2_ thin films can take place on quartz (SiO_2_) substrates. However, in the latter case, the α–β phase transition that takes place in both substrates and thin films during heating deteriorates the long-range order and, thus, the piezoelectric properties. Here, we report the ousting of spherulitic growth by using a buffer layer. Using TiO_2_ as a buffer layer, the epitaxial strain of the substrates can be transferred to the growing films, leading to the oriented crystallization of GeO_2_ in the α-quartz phase. Moreover, since the TiO_2_ separates the substrates and the thin films, the thermal stability of the GeO_2_ is kept across the substrate’s phase transitions. Our findings reveal the complexity of the crystallization process of quartz thin films and present a way to eliminate the tendency for spherulitic growth of quartz thin films by epitaxial strain.

## Abstract 2

The crystallization of epitaxial thin films of GeO_2_ in the α-quartz phase has been achieved through the use of a TiO_2_ buffer layer on α-quartz (SiO_2_) substrates. This works shows that it is possible to avoid the formation of spherulites with randomized orientation, which are detrimental to the piezoelectric properties of the films

## Introduction

α-Quartz (SiO_2_) is one of the most used piezoelectric materials. Its crystal structure has a trigonal symmetry with space group *P*3_1_21 (left-hand) or *P*3_2_21 (right-hand) where the *c*-axis [0001] is the 3-fold axis.^1^ Besides SiO_2_, GeO_2_ can also crystallize into the α-quartz phase, with a larger lattice: *a* = 4.989 Å and *c* = 5.653 Å, compared to *a* = 4.916 Å and *c* = 5.408 Å of SiO_2_.^2^ GeO_2_ also has higher piezoelectric coefficients (*d*_11_ = 6.2 ± 0.3 pC/N and *d*_14_ = 2.7 ± 0.5 pC/N,^3^ compared to *d*_11_ = 2.31 pC/N and *d*_14_ = 0.73 pC/N of SiO_2_^4^), higher estimated piezoelectric constants,^5^ and better thermal stability than SiO_2_. At 573 °C, α-quartz transforms into β-quartz, with hexagonal symmetry.^6^ As a result of that, *d*_11_ is lost and only *d*_14_ remains. On the contrary, for bulk single-crystal GeO_2_, the α–β phase transition is absent, and α-quartz is the only phase present until its melting at 1116 °C.^7^

In our previous study, we have grown thin films of GeO_2_ on various substrates: Al_2_O_3_, MgAl_2_O_3_, MgO, LaAlO_3_, and SrTiO_3_, with crystal lattices quite different from that of quartz. These thin films are crystallized into the α-quartz phase in the form of fully relaxed spherulites, in which the lattice rotates linearly with the distance to the nucleation center, as the growth progresses.^8,9^ Spherulites are crystal ensembles that generally have spherical (in three dimensions) or circular (in two dimensions) shapes and are composed of fibers that grow radially from the nucleation center. Spherulites are often found in materials with poor crystallinity, such as polymers,^10,11^ liquid crystals,^12^ geological minerals,^13^ and so on. The formation of spherulites is dominated by noncrystallographic branching, where new branches grow with small misorientation with respect to the parent crystals.^14^ As a result of this lack of orientation, spherulites cannot be used in piezoelectric applications.

To overcome this issue, epitaxial thin films have been grown. If the material is grown as a thin film on a single-crystal substrate, and the substrate and thin-film crystal lattices are sufficiently similar to each other, the thin film can grow epitaxially and become strained by the substrate. The strain arises from the lattice mismatch, and it is calculated as ϵ = (*a*_s_ – *a*_f_)/*a*_f_, where *a*_s_ and *a*_f_ are the bulk lattice parameters for the substrate and thin film, respectively. We have reported that, when the GeO_2_ is deposited directly on quartz (SiO_2_) substrates, due to the small lattice mismatch with the same crystal structure, the thin films grow epitaxially, with the same orientation as the substrates.^15^ However, when the sample is heated at 573 °C, the thin films transform into the β-quartz phase following the substrate,^15^ again leading to the disappearance of the longitudinal piezoelectric response associated with the *d*_11_ component of the piezoelectric tensor.

In this study, we use TiO_2_-buffered quartz (SiO_2_) substrates, where the TiO_2_ layer transfers the strain from the substrates into the GeO_2_ thin films. In this way, we are able to achieve epitaxial GeO_2_ thin films avoiding the spherulite formation that takes place on nonquartz substrates, with the same growth conditions. Moreover, the TiO_2_ layer provides a sufficiently different crystal structure to prevent that the GeO_2_ layer follows the α–β phase transition of the substrate, thus maintaining the thermal stability of GeO_2_. Our results suggest that, besides the known factors determining the formation/disappearance of spherulites, such as the temperature^16,17^ and composition of alloy,^18^ strain can be used to prevent spherulitic growth.

## Experimental Section

Thin films of GeO_2_ with a buffer layer of TiO_2_ were deposited by pulsed laser deposition (PLD) using a 248 nm KrF laser (Lambda Physik COMPex Pro 205). For the GeO_2_ target, first the GeO_2_ powder (Alfa Aesar, 99.9999%) was milled for 90 min at 150 rpm and then the fine powder was pressed into pellets with cold pressing at 10 tons. Finally, the pellets were sintered at 900 °C for 1 h. The TiO_2_ target was made by a similar procedure with TiO_2_ powder (Alfa Aesar, 99.6%). The milling condition was 3 h at 200 rpm, and the sintering was done at 1100 °C for 4 h.

First, a layer of TiO_2_ was deposited, the deposition parameters were the following: target–substrate distance of 52 mm, fluence of 2 J/cm^2^, laser repetition rate of 1 Hz, oxygen pressure of 0.1 mbar, and deposition temperature of either 600 or 830 °C. The substrates were heated by an infrared laser (DILAS Compact-Evolution; wavelength: 808 nm) during the growth. After the deposition of TiO_2_, GeO_2_ was deposited with a target–substrate distance of 46 mm, fluence between 2.5 and 3.5 J/cm^2^, and oxygen pressure of 0.1 mbar. For the GeO_2_ deposited at 600 °C, the laser repetition rate was 5 Hz, while for the GeO_2_ films deposited at 830 °C, the laser repetition rate was 2 Hz. After the deposition, some of the films were annealed in 200 mbar of oxygen at 730 or 830 °C for different times (as described later). Afterward, the films were cooled down to room temperature with a rate of −5 °C/min.

The growth process was monitored in situ by reflection high-energy electron diffraction (RHEED). The local crystallinity and crystal orientations were characterized by electron back-scatter diffraction (EBSD), performed on a FEI Nova NanoSEM 650 scanning electron microscope with the sample pretilted on a 71° holder. To minimize the charging effect from the insulating substrates and thin films, the chamber pressure was kept at 0.5 mbar, and a low-vacuum detector was used. EBSD data was collected using an energy-dispersive X-ray analysis (EDAX) EBSD system equipped with a Hikari CCD (CCD = charge-coupled device) camera for recording Kikuchi patterns. EDAX OIM Analysis 8.1 and MATLAB-based toolbox MTEX^19^ software were used for EBSD data analysis. Inverse pole figure (IPF) and image quality (IQ) maps were plotted along three axes [100], [010], and [001] in Cartesian coordinates, where [001] is the out-of-plane direction of the sample and [100] and [010] are in-plane directions, the [100] direction being horizontal on the maps. The average crystallinity was checked by X-ray diffraction (Panalytical X’Pert, CuK_α_ radiation).

The XRD reciprocal space mapping (RSM) was done by a Bruker D8 Discover diffractometer with a high-brilliance microfocus Cu rotating anode generator and an EIGER2 R 500 K area detector. The measurements were done with a 100 μm diameter circular pinhole beam collimator to shape the beam. The surface morphology was imaged by atomic force microscopy (AFM) (with a Bruker Dimension Icon microscope) in tapping mode and ScanAsyst mode.

**Figure 1:**
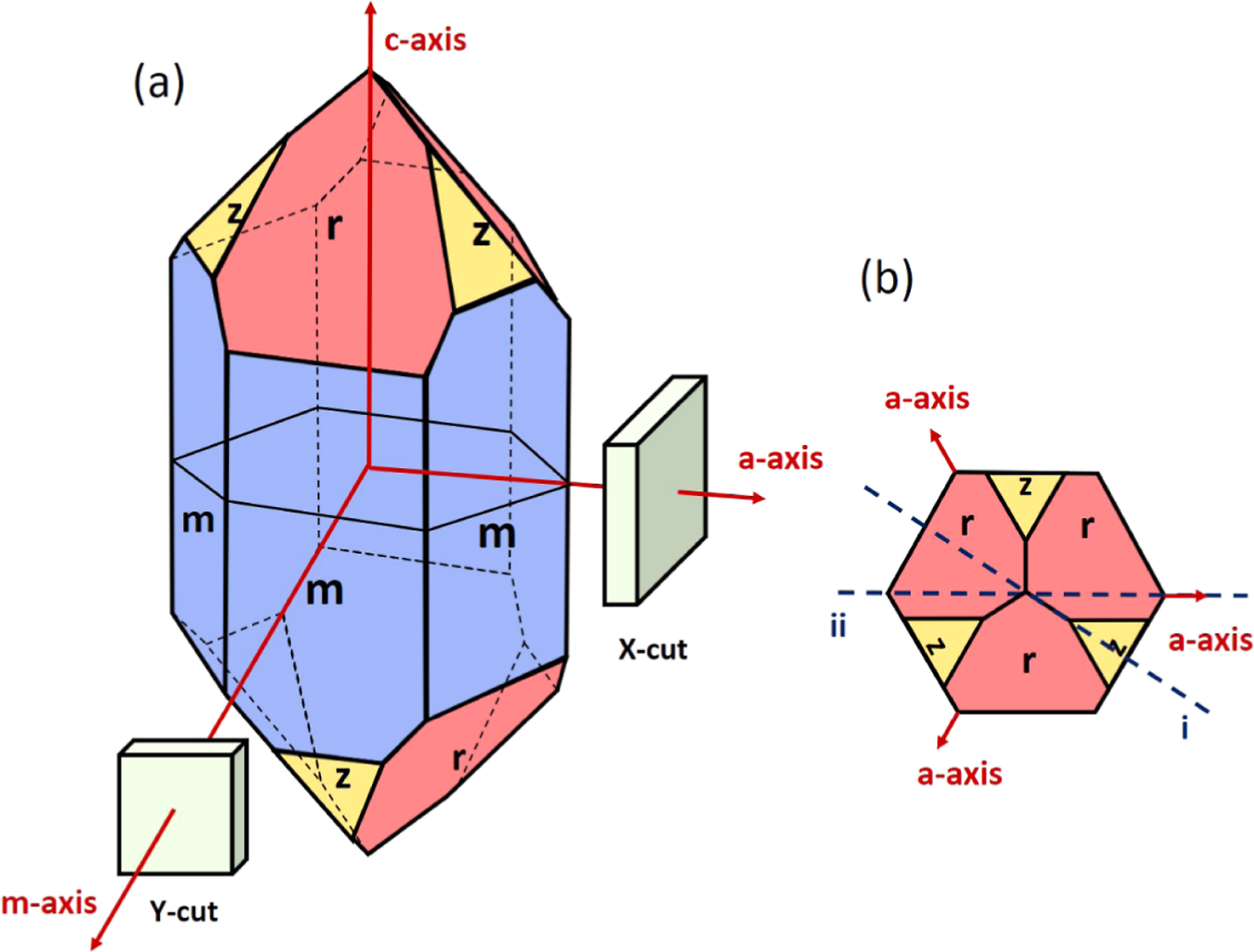
(a) Schematic of the quartz crystal with an idealized shape. (b) Projection along the *c*-axis of the crystal in (a). (i) In-plane orientations of the leaf crystal in Figure 3a. (ii) In-plane orientations of the leaf-shaped crystal in Figure 3b.

**Figure 2:**
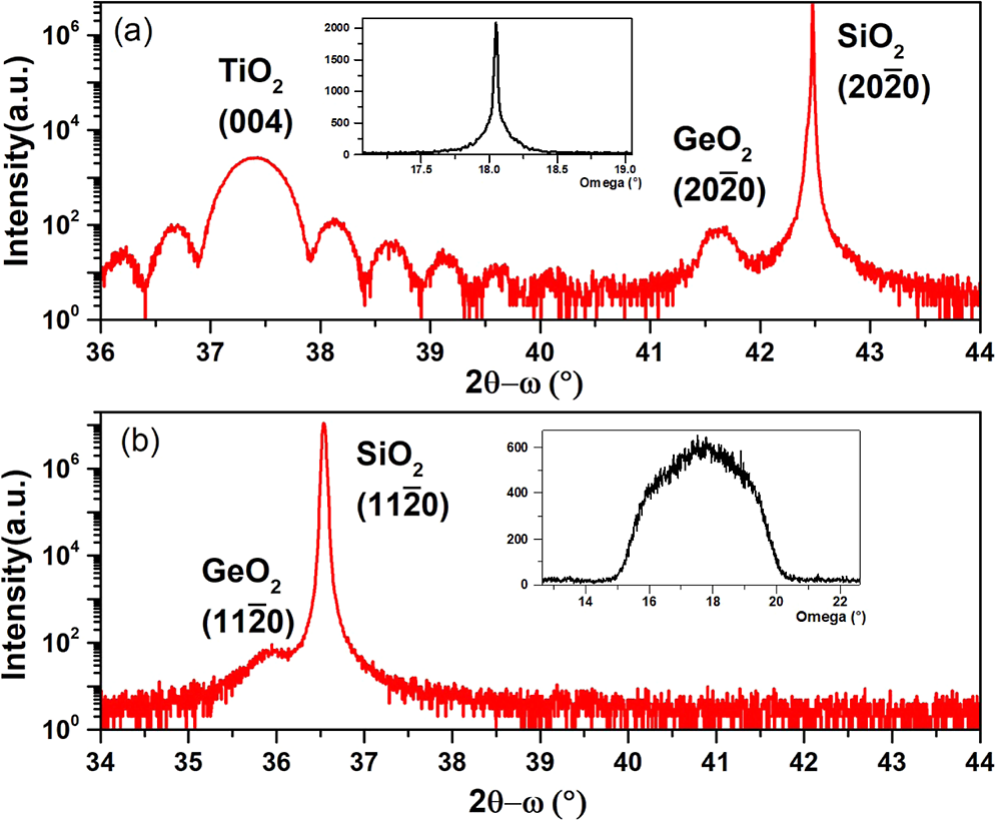
2θ–ω scan of the thin films on Y-cut substrates (a) and X-cut substrates (b). Inset panels: (a) rocking curve of TiO_2_ (004) and (b) rocking curve of GeO_2_.

The substrates used in this study were 5 × 5 mm^2^ X-cut  and Y-cut  quartz (SiO_2_) substrates (CrysTec GmbH). In Figure 1, we show a sketch of a quartz crystal, indicating the orientations of the crystallographic axes and these cuts. α-Quartz has a trigonal symmetry with space group *P*3_1_21 (left-hand) or *P*3_2_21 (right-hand), where the *c*-axis [0001] is the 3-fold axis.^1^ It has six equivalent prismatic faces, , denoted by “*m*”, and , which we denote by “*m*-axes”, are the corresponding directions perpendicular to these prismatic planes. Due to the trigonal symmetry of α-quartz, the “*a*^+^” direction, , and “*a*^–^” direction, , are not the same. For the X-cut substrates, the out-of-plane direction is the *a*-axis, with the two in-plane orthogonal directions being the *c*-axis and *m*-axis. For the Y-cut substrates, the out-of-plane direction is the m-axis and the two in-plane orthogonal directions are the *c*-axis and *a*-axis. Figure 2. The crystallographic orientation of these two cuts is drawn in Figure 1a.

## Results and Discussion

### Growth of the TiO_2_ Buffer Layer

To characterize the piezoelectric response of the thin films (awork in progress), it is necessary to separate the thin film and the substrate. For that, a layer of TiO_2_ with the thickness of about 18 nm was grown on X-cut  and Y-cut  quartz substrates. The in situ RHEED (Figure S1) shows grid patterns that indicate the crystalline and oriented nature of the film. AFM scans (see Figure S1) reveal that in both cases TiO_2_ crystallizes into small rectangular grains with a width of about 50 nm. Figure 2a shows the 2θ–ω scan of a GeO_2_ film on a TiO_2_-buffered Y-cut quartz substrate. In this section, we focus our discussion on the TiO_2_ layer, which shows a peak at 37.4° that corresponds to the (004) of the tetragonal anatase phase of TiO_2_.^20^ The presence of Laue oscillations indicates good crystallinity of the TiO_2_ layer. The out-of-plane lattice parameter is extracted from the 2θ–ω scans and found to be *d*_001_ = 9.608 Å, which is larger than the bulk lattice *d*_001_ = 9.514 Å.^20^ A rocking curve of the TiO_2_ (004) peak, which has a FWHM (full width at half-maximum) of 0.04 deg (see inset in Figure 2a), again confirms the good epitaxial quality of this layer.^21^Figure S2a in the Supporting Information shows a 2θ–ω scan for the TiO_2_ thin films on X-cut substrates, with a larger scan range from 33 to 80°, showing the (117) reflection of the anatase phase as the out-of-plane direction.

The in-plane orientation of TiO_2_ is revealed by EBSD (see Figure S2b). For the TiO_2_ grown on Y-cut substrates, a single epitaxial relationship was found: the TiO_2_ is oriented with (001) out-of-plane, ⟨110⟩ (*d* = 5.351 Å) align with the [0001] (*d* = 5.408 Å), and  (2*d* = 4.916 Å) of quartz. Thus, the TiO_2_ is under compression along one of the in-plane directions; while it is under tension in the orthogonal direction. Since the mismatch with  is larger, in general, TiO_2_ is under in-plane compression, leading to the observed expansion in the free out-of-plane direction. On the other hand, TiO_2_ grown on X-cut substrates fits the substrates with two orientations: () or () out-of-plane, with [110] aligning parallel to [0001] of the substrate. However, in this case, the lattice does not have a close match in the orthogonal in-plane direction, with *d*_771_ = 5.947 Å, in comparison with .

### Epitaxial Quartz Crystals on TiO_2_-Buffered Quartz Substrates

**Figure 3:**
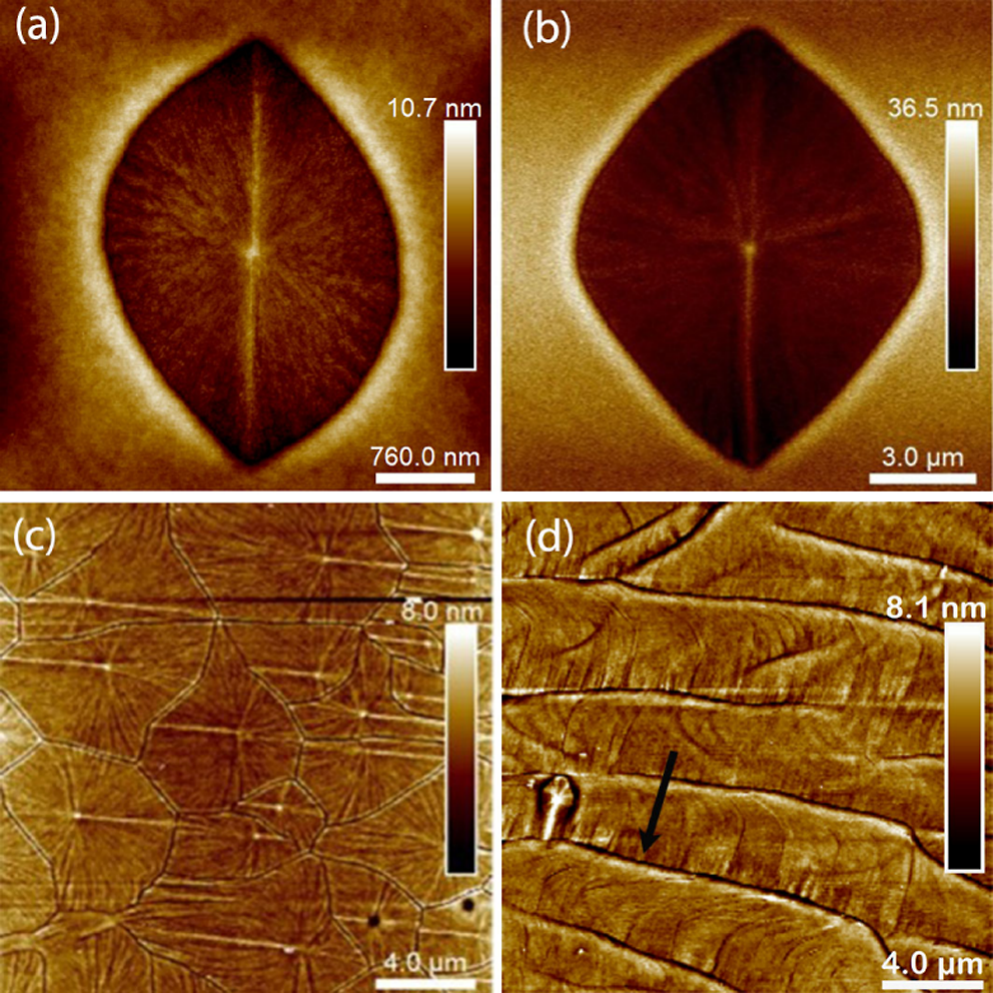
AFM images of leaf-shaped GeO_2_ quartz crystals: (a) on an X-cut substrate; (b) on a Y-cut substrate; (c) clustering to form a Voronoi-like tessellation pattern, on the X-cut substrate; (d) embedded in semiperiodic wave-like structures, on the Y-cut substrate. The black arrow points to the crystallization direction of the wave-like structures.

After the growth of the TiO_2_ layer, a layer of GeO_2_ was deposited on top of it at 830 °C with a laser repetition frequency of 2 Hz. Generally speaking, the crystallization of quartz on X-cut and Y-cut substrates is very similar. 2θ–ω scans in Figure 2 have shown that GeO_2_ has crystallized into the quartz phase for both cuts. Figure 3a,b shows the GeO_2_ quartz crystals surrounded by the amorphous GeO_*x*_, on X-cut and Y-cut, respectively. Both crystals have a leaf-like shape with a core in the center serving as the nucleation point, from which fibers grow radially. These crystals are lower in height compared to the amorphous surroundings, as expected due to the densification taking place upon crystallization, and they display a halo that indicates accumulation of material at the crystal edge. The main difference in crystallization between the films on X-cut and Y-cut quartz is the nucleation rate. On X-cut substrates, the nucleation rate is significantly higher than that of films on Y-cut substrates. As a result of that, after cooling to room temperature, the thin films on X-cut substrates are crystallized completely, while those on Y-cut substrates are only partially crystallized. However, additional 30 min of annealing at the deposition temperature can help the thin films on Y-cut to complete crystallization.

When the nucleation rate is high, as in the thin films on X-cut substrates, multiple leaf crystals nucleate and grow, filling the space, as shown in Figure 3c. When the nucleation rate is low, as in the thin films on Y-cut substrates, a different type of crystals composed of fibers and forming semiperiodic wave-like structures compete with the crystallization of the leaf crystals, as shown in Figure 3d. These fibrous crystals, which are also in the quartz phase, start at multiple nucleation points at the edge of the sample and then expand continuously toward the sample center. It can be seen that each period is separated by a dip in height, followed by a tall rim where the material accumulates. Small fibers grow out from the rim and end almost perpendicular to the dip. (See Figure S3 in Supporting Information for the height profile.) We have observed similar wave-like structures in GeO_2_ thin films on Al_2_O_3_, MgO, LaAlO_3_, and SrTiO_3_ substrates.^8^ Moreover, the leaf crystals can be embedded in the wave structures. As shown in Figure 3d, this leaf crystal has an elongated tail due to the sweeping of the crystallization of the wave structures, with the direction of the tail pointing along the crystallization direction of these structures, i.e., perpendicular to the waves, or along the fibers, as pointed by the black arrow. We have found that these wave-like structures are easier to relax than the leaf crystals.

**Figure 4:**
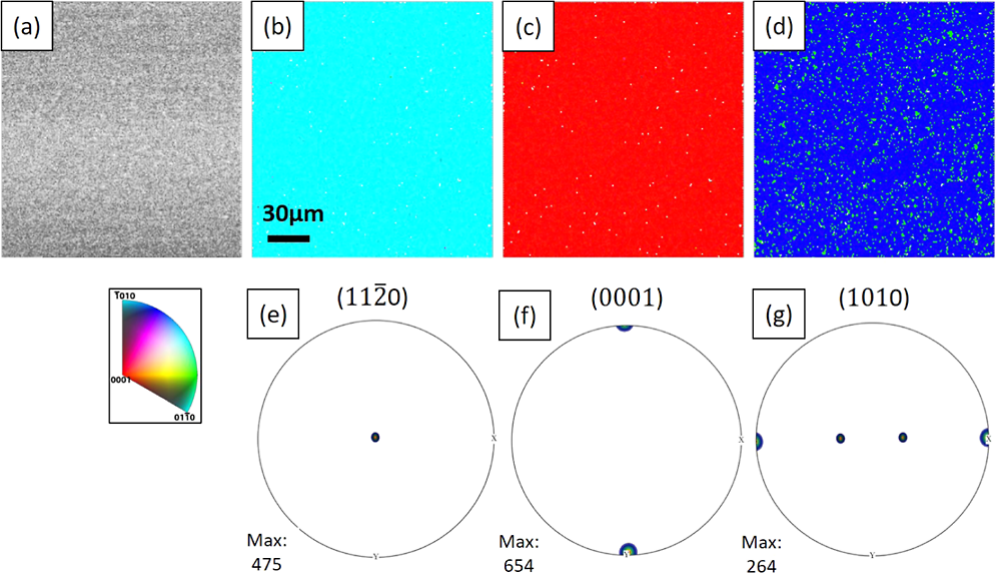
EBSD analysis of a GeO_2_ thin film on a TiO_2_-buffered X-cut substrate. (a) IQ map. (b–d) IPF maps viewed from [100], [010], and [001] directions of the sample, respectively, showing that the thin film has the same orientation as the substrate. (e–g) Pole figure density plots for , (0001), and  poles, respectively, showing the strong texture of the film.

Figure 4 shows the EBSD result of the GeO_2_ thin film grown on the X-cut, corresponding to Figure 3c. Figure 4b–d shows the IPF maps, where the colors represent different crystal planes viewed from the [100], [010], and [001] directions of the sample, respectively. It is clear that all three IPFs show a strongly preferred orientation, which is also quantitatively reflected in the pole figures in Figure 4e–g. These figures show that the thin films share the same orientation of the substrates, with the  out-of-plane and in-plane *c*-axis of the thin film aligning with the *c*-axis of the substrates. Figure 4b–d displays a homogeneous color, while for Figure 4d green pixels  can be observed from the blue background . In fact, these blue and green areas approximately match the Dauphiné twinning. Dauphiné twinning is twins related by a 180° rotation around the *c*-axis, detected by EBSD as a 60° misorientation around [0001] due to the trigonal symmetry of quartz. This twinning does not change the chirality of the crystal, but it reverses the polarity of the *a*-axis, which leads to the cancelation of the piezoelectric effect. The strong epitaxial relationship observed in the films shows that the TiO_2_ layer is able to transfer the strain from the substrate. As expected, the TiO_2_ layer can transfer the lattice spacing, but it cannot transfer information on the substrate polarity/chirality, resulting in both *a*+ and *a*– orientations (twins). This confirms that the strong preferred orientation is induced by epitaxy from the substrates through the buffer layer.

**Figure 5:**
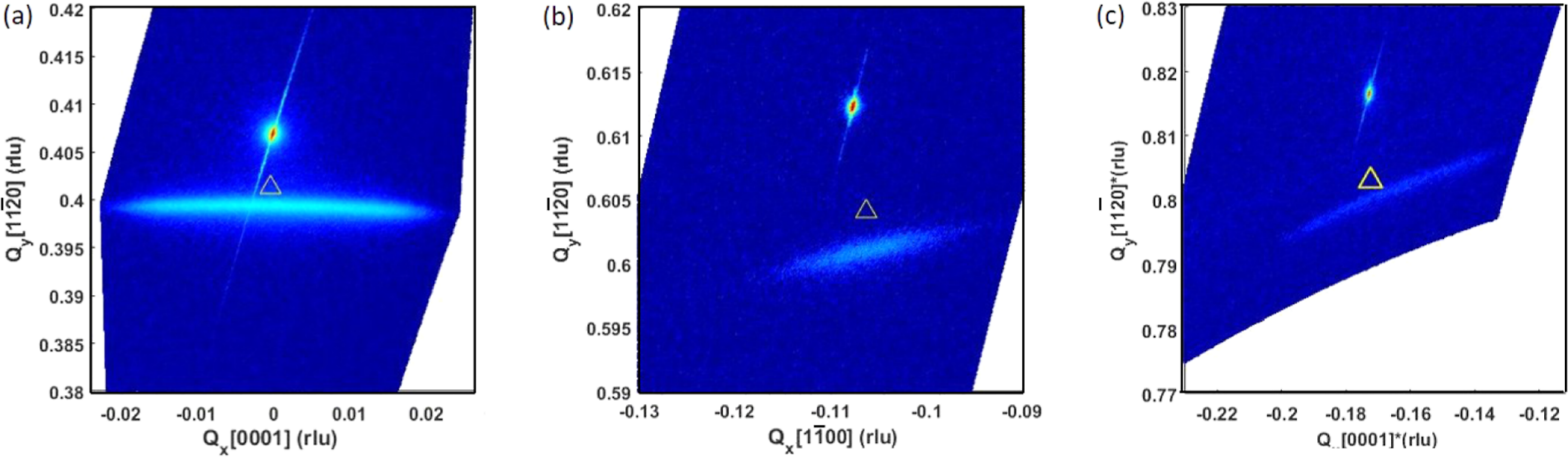
RSM of a GeO_2_ thin film on the TiO_2_-buffered X-cut substrate: (a) specular , (b) nonspecular , and (c) nonspecular . The triangles represent the positions of the bulk GeO_2_ lattice. All film peaks show a significant amount of in-plane spread, indicating the relaxation of the crystals during crystallization.

Although EBSD shows the GeO_2_ thin film has a strong texture with the same orientation as the substrate, RSMs by XRD reveal that the GeO_2_ lattice has relaxed in the in-plane direction. As shown in Figure 5, it is clear that all the film peaks have a significant amount of in-plane spread, both in  and [0001] directions. On the other hand, this peak broadening is absent in the out-of-plane direction. A rocking curve of the specular peak  (Figure 2b inset) shows a large FWHM of about 3.9°. This suggests the presence of a significant number of defects in the film, such as dislocation and lattice bending. The GeO_2_ peak positions corresponding to the bulk lattice are marked with triangles in Figure 5. The in-plane spread makes it difficult to calculate the lattice parameter. However, for the out-of-plane, it is clear that the lattice is shifted away from the bulk peak, with the expansion of  from 2.491 Å from the bulk to 2.506 Å, which is about 0.6% expansion.

**Figure 6:**
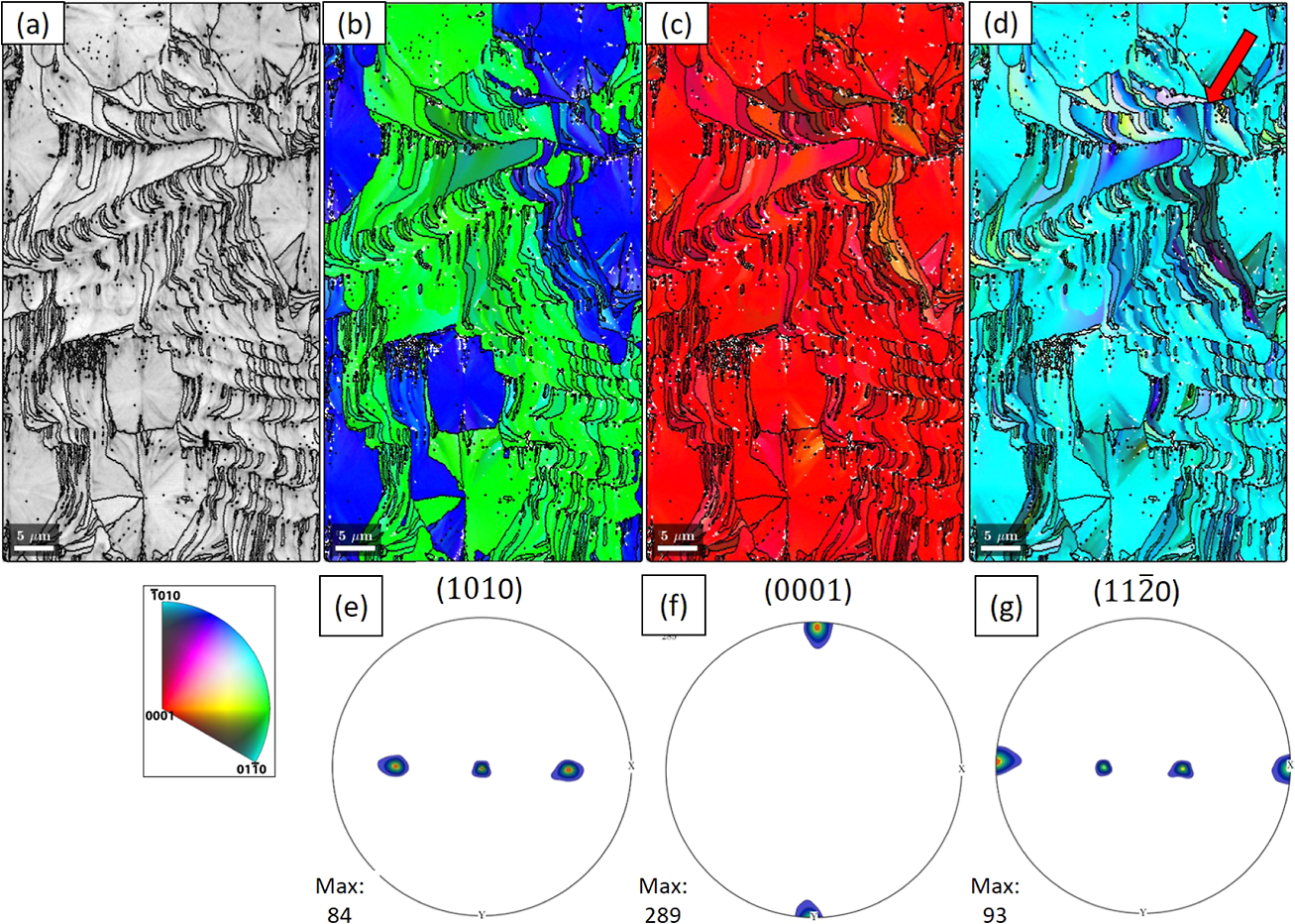
EBSD analysis of a GeO_2_ thin film on the TiO_2_-buffered Y-cut substrate (a) IQ map. (b–d) IPF maps viewed from [100], [010], and [001] directions of the sample, respectively, showing that the thin film has the same orientation as the substrate, with local relaxation at some places (mostly in the wave structures). The black lines in each IPF are the grain boundaries with the misorientation angle larger than 3°. The propagation of the wave structures runs from the bottom to the top of the figure. (e–g) Pole figure density plots for , (0001), and  poles, respectively.

Similar to GeO_2_ films grown on X-cut, films on Y-cut substrates also grow with a strongly preferential out-of-plane orientation aligned with the substrate. Figure 6a shows the IQ map of an area of GeO_2_ on TiO_2_-buffered Y-cut substrates, reflecting the crystallinity of the thin film. Any features disrupting the periodicity of the lattice, including grain boundaries, strain, dislocations, etc., will result in a dark contrast in the map. It can be observed that the IQ map also reflects the morphology of the films, as shown in Figure 3d, where the leaf-shape crystals and the surrounding wave structures are clearly visible. The IPFs from Figure 6b–d clearly prove the preferential orientation of the films. As mentioned earlier, the () (blue) and () (green), corresponding to the *a*^+^ and *a*^–^ axes, respectively, match with the Dauphiné twinning of each other (see the EBSD map of twinning in Figure S4).

In some parts of Figure 6b–d, a gradual color change is observed, evidencing local lattice relaxation. Surrounding the leaf crystals, semiperiodic wave structures can be observed,^8^ and the grain boundaries depict clearly the growth and branching of the fibers. Figure 6a shows that, in each period, the fibers first grow curved and then change their direction to be perpendicular to the wave fronts, creating the war-horn shape of the grain. This is also reflected in the morphology, as described earlier for Figure 3d. Usually, multiple fibers are contained in one grain (which is chosen as the region with misorientation angles between fibers smaller than 3°, as mentioned earlier). At some periods, as pointed out by the arrows in Figure 6d (also see S5 in Supporting Information for another example with higher magnification), the beginning of the wave has a homogeneous orientation, while later it branches into a group of nearly parallel fibers with different orientations. In some cases, the misorientation between the new fibers is small enough such that they are still considered as part of the same grain. This relaxation is reflected also in the pole figures in Figure 6e–g, where all the poles are more smeared and the maximum density is smaller compared to films on X-cut.

Similarly, RSMs of films on the Y-cut also have an in-plane spread as on the X-cut, but with weak intensity, probably due to more spread of the film orientation. The out-of-plane lattice parameter is extracted from the 2θ–ω scan with  = 4.336 Å, which is comparable to GeO_2_ directly grown on SiO_2_ quartz substrates  = 4.331 Å.^15^ The out-of-plane expansion is induced by in-plane strain from the substrate.

**Figure 7:**
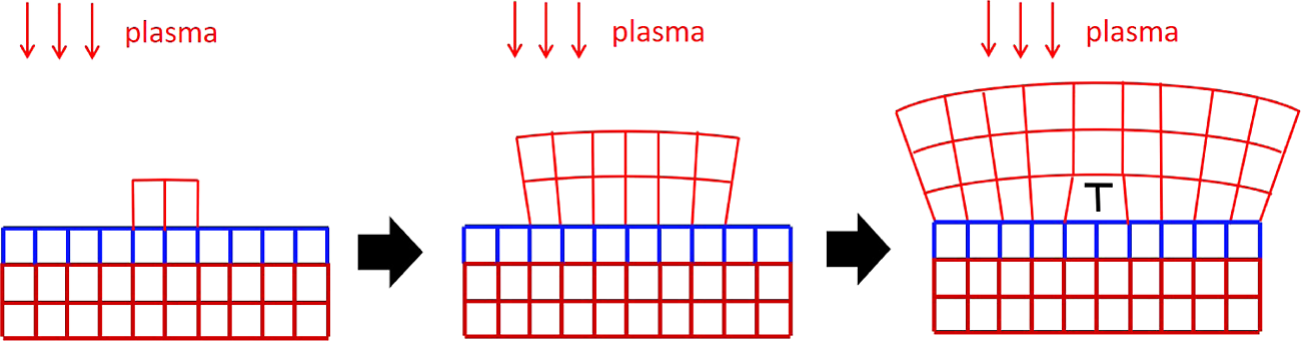
Schematic diagram of the crystallization of GeO_2_. Red: GeO_2_ lattice, blue: TiO_2_ lattice, and dark red: SiO_2_ substrate lattice. As the crystallization expands laterally, stress accumulates, and the film is relaxed by forming dislocations and by lattice rotation.

Figure 7 presents a schematic diagram of the crystallization of GeO_2_. The TiO_2_ layer grows epitaxially on the quartz substrate, transferring the strain from the substrate to GeO_2_ on top. Because of the different phases between GeO_2_ and TiO_2_, nucleation is necessary for the crystallization to occur. Due to the strain from the substrate, the nuclei form epitaxially with the substrates. As a result of in-plane stress, the out-of-plane lattice is expanded slightly. When the plasma reaches the crystalline part, it can readily crystallize into the quartz phase. However, if it reaches the amorphous part, it still has to diffuse and reorientate to attach to the growing crystal, or the other way round the crystal expands laterally. As the crystal keeps growing, the stress accumulated in the crystal increases, and it becomes harder and harder for the substrate to strain the film. Thus, the film starts to relax, which results in the spread we observed in XRD and rotation in EBSD. Unlike the leaf crystals, where the size is often limited to several μm, the wave-like crystals can grow continuously across the film, which suggests more stress accumulation. This leads to more lattice rotation and explains the difference in films on X- and Y-cut substrates.

It is also worth noting that the shape of the leaf crystals reflects the orientation of the crystal and the symmetry of quartz. From the EBSD analysis, we know that the long axis of the leaf crystal is the *c*-axis. This suggests the *c*-direction being the fastest growth direction, as for the bulk quartz crystal.^22,23^ Moreover, the leaf shape of the crystal resembles the computer-simulated single-crystal growth shape.^22^ In addition, looking closer, it can be seen that the crystals on Y-cut substrates (Figure 3b) are symmetric with respect to the long axis, while this is not the case for the crystals on X-cut in Figure 3a (see also S6 in Supporting Information), suggesting that the growth speed varies in adjacent regions (i.e., growth speed at the top-left quarter of Figure 3a is different from that of the top-right quarter but similar to that of the bottom-right quarter).

These can be explained by the trigonal symmetry of the α-quartz phase. As shown in Figure 1, the α-quartz crystal has three major rhombohedron faces, denoted “*r*”, and three minor rhombohedron faces, denoted “*z*” (in this paper, we use the *r* settings where the *r*-faces are indexed with  and *z*-faces are indexed with (1,24)). In bulk single crystals, usually the *r* faces are larger than the *z* faces. This different area size results from the differences in the growth rate perpendicular to these faces (faces with a smaller growth rate as expected to have a larger surface area). For the leaf crystals on the X-cut substrates, as shown in the cross-sectional plane (i) in Figure 1b, on the one side is the major rhombohedron *r*-face, while the other side is the minor rhombohedron *z*-face. On the contrary, for the leaf crystals on the Y-cut substrates, both sides display the large rhombohedron *r*-faces, as shown in the cross-sectional plane (ii).

The lack of symmetry of leaf crystals on X-cut substrates suggests that, during deposition, GeO_2_ grows in the α-quartz phase, even though the SiO_2_ substrate is in the β-quartz phase. Indeed, the buffer anatase TiO_2_ layer only transfers the lattice information from the substrate, while other information related to the chemistry, such as symmetry and handness, is lost. On the contrary, as we have found in our previous work, when the GeO_2_ is grown directly on SiO_2_ quartz substrates, it will follow the α–β phase transition of the substrates, losing *d*_11_ in the β-phase at 573 °C.^15^

### Competition between Epitaxial Growth and Spherulitic Growth

However, in some cases, GeO_2_ can crystallize into spherulites locally by bypassing the strain from the substrates. In this case, the film is deposited at a lower temperature of 600 °C with a 5 Hz laser repetition rate and then annealed at various temperatures with a heating up rate of 25 °C/min. After deposition, the surface of the film is amorphous due to a lower thermal budget; in this way, it is possible to crystallize from the film surface. Meanwhile, the epitaxial nuclei are able to form at the TiO_2_–GeO_2_ interface.

When the thin film is annealed at 730 °C for 30 min, the thin film is only partially crystallized. As shown in Figure S7 (see Supporting Information), only the edge is crystallized, and there is a gradient of the number of leaf crystals, which decreases when moving toward the center of the sample. We have also observed the same promotion of nucleation at the edge in GeO_2_ thin films on Al_2_O_3_ substrates.^8^ In this case, the spherulitic fibers have not sprouted yet.

By comparing the density of the leaf crystals in the center of the sample, as shown in Figure S7 (see Supporting Information), we can conclude that the nucleation rate decreases dramatically from 830 to 730 °C. However, their leaf-like shape tells that the crystals are still epitaxial. Moreover, the nonsymmetrical shape again confirms their epitaxial orientation, as described in the previous section.

If the thin film is annealed for a longer time (2 h), more of the material is able to crystallize, as shown in Figure S7 (see Supporting Information). However, the sizes of the leaf crystals are comparable to the ones with shorter annealing, which suggests that further growth of the leaf crystals is limited, most likely by the strain from the epitaxial growth. On the contrary, as shown in the optical images in Figure S7 (see Supporting Information), with a longer annealing time, the spherulitic fibers have sprouted from the edge and expand continuously to occupy the rest of the space. The clear arc shape of its crystallization front reflects the radial growth of the fibers starting from the sample edge. We can estimate the growth speed from the expansion to be about 5 μm per minute at 730 °C.

**Figure 8:**
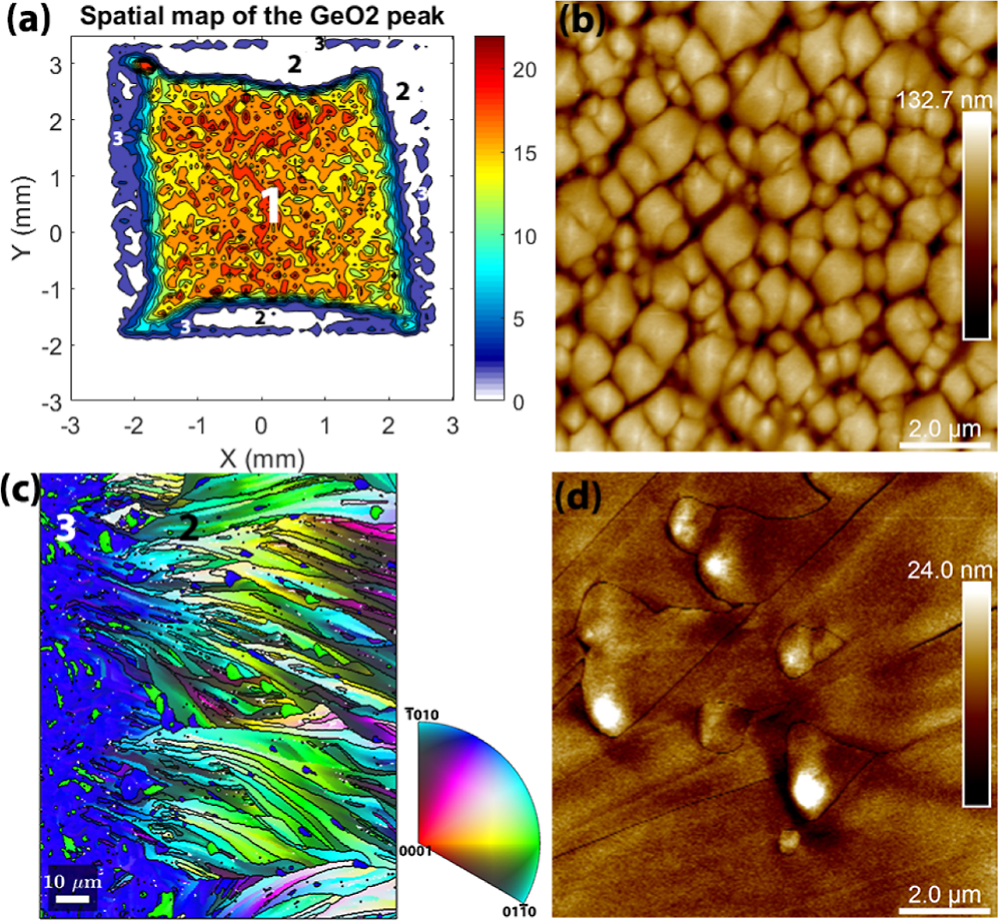
Thin films of GeO_2_ grown on the X-cut substrate, deposited at 600 °C with a 5 Hz laser deposition rate and annealed at 830 °C for 30 min. (a) Spatial mapping of GeO_2_*a*+  and *a*–  peaks and the three areas in which the map can be divided (as shown by the numbers), according to the magnitude of the XRD intensities. (b) AFM scan of Area 1 shows that it is composed of dense quartz leaf crystals. (c) IPF image at the edge of the sample viewed from the [001] direction shows the epitaxial Area 3 and spherulitic Area 2 with clear color gradient in the fibers. (d) AFM scan of Area 2 shows leaf crystals with an elongated tail embedded in the nonoriented branches.

When we raised the annealing temperature to 830 °C, the whole film is crystallized. Figure 8a shows the mapping of the  and  peaks (they both contribute to the intensity of XRD, as they share the same 2θ angle) across the entire film surface (5 × 5 mm) of the film annealed at 830 °C for 30 min, showing an overview of the degree of crystallinity as a function of the position on the sample. Unlike the films in the previous section, the film can be divided into 3 regions as marked in Figure 8a: Area 1, the center part, which gives the strongest XRD intensity; Area 3, at the very edge of the sample, which gives weak intensity, and Area 2, the region in between Area 1 and Area 3, that gives the lowest intensity. These three regions are also clear under the optical microscope in Figure S8 (see the Supporting Information).

Figure 8b shows the AFM scan at Area 1, the center of the film, showing that it is filled with a large amount of quartz leaf crystals, typically less than 1 μm in diameter. EBSD has proven that all the leaf crystals are oriented equal to those of thin films grown at 800 °C on X-cut substrates previously discussed; i.e., it grows epitaxial with the same orientation as the substrate. Figure 8c shows the local orientation of the boundary between Area 2 and Area 3. It is clear that Area 3 (the very edge of the sample) has the same epitaxial relationship as Area 1. On the contrary, Area 2 shows a typical spherulitic growth. Further analysis (see Figure S9 in Supporting Information) shows that the lattice rotates linearly with its growth distance in one fiber, and from statistical analysis a typical rotation angle gradient is found to be about 0.6–1.25°/μm.

This explains why there is almost no signal from Area 2 for the XRD mapping. The phenomenon of lattice rotation is also observed and studied systematically in our previous study.^9^ Interestingly, a closer examination at Area 2 in Figure 8c shows some blue and green semicircular pixels in between the long fibers. AFM scans shown in Figure 8d reveal that they are leaf crystals but distorted due to the sweeping of these spherulitic fibers: the crystals have an elongated tail, which points to the fiber growth direction. Although these quartz crystals are shaped by the fibers that have various orientations, they are still epitaxial as in Area 1 and Area 3.

**Figure 9:**
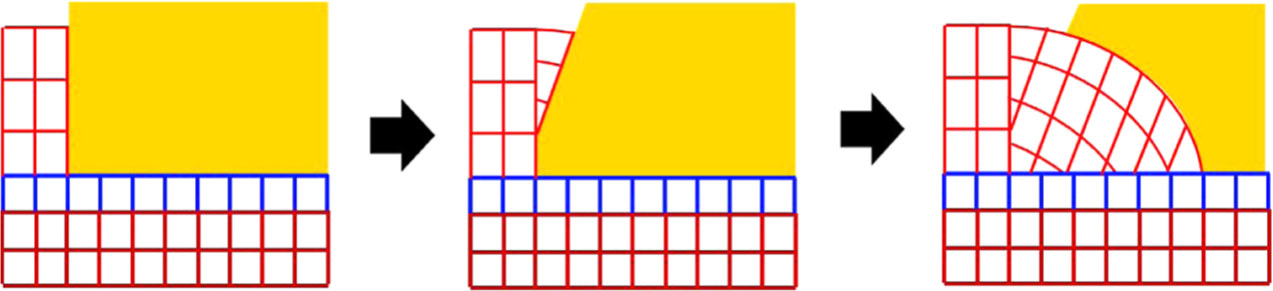
Schematic diagram of the crystallization of GeO_2_. Red: GeO_2_ lattice, blue: TiO_2_ lattice, dark red: SiO_2_ substrate lattice, and yellow: amorphous GeO_2_. This figure depicts the case in Figure 8 Area 3, where spherulitic fibers grow out of the epitaxial area. Since the crystallization is spherulitic, it does not have to match the existing lattice.

As it is apparent, nucleation kinetics is of vital importance in the crystallization process. Taira et al. have reported lateral solid-phase epitaxy of TiO_2_ on the glass substrate using nanosheets.^25^ When the first layer of TiO_2_ is thin, oriented TiO_2_ is able to form on top of the nanosheets, while the rest of the film is still amorphous. Later, these nuclei will grow laterally and form an epitaxial film. However, if the first layer of TiO_2_ is thick, although oriented nuclei can still form on the nanosheets, the rest of the TiO_2_ deposited directly on glass substrates nucleated in randomly oriented crystals that cannot be further reoriented. This is similar to the competition between epitaxial and spherulitic crystallization in this study: whether it will crystallize into oriented grains or spherulites depends on which type of nuclei are formed. Figure 9 shows spherulitic crystallization. Unlike epitaxial growth in Figure 7, the nuclei formed at the film’s top due to local kinetics, i.e., deposition conditions, defects, local stress, and so on. Without the strain from the substrates, at this temperature, the nuclei will grow into spherulitic fibers as we have observed in our previous study.^8,9^

## Conclusions

In this study, we have successfully grown epitaxial thin films of α-quartz GeO_2_ on TiO_2_-buffered X-cut and Y-cut quartz (SiO_2_) substrates. The epitaxial stress from the substrates, which is transferred to the film through the buffer layer, is a determinant factor in the oriented growth of the thin films. Without it, at the annealing temperature of 830 °C, the GeO_2_ films crystallize into spherulitic quartz. High temperatures and low deposition rates facilitate epitaxial growth; while low temperatures and high deposition rates favor spherulitic growth. In an amorphous area, the nucleation occurs at the interface between the TiO_2_ layer and the amorphous thin film, followed by crystallization from the bottom of the film to the top of the film, resulting in epitaxial leaf crystals. At the edge of the sample, the nucleation rate is significantly enhanced and the edge is fully crystallized earlier than the rest of the film. This creates a growth front where secondary nucleation can occur at the perimeter of the existing quartz crystals. With the continuous nucleation and growth, wave-like structures form and the crystallization continues sweeping toward the center of the sample. When the temperature is high and the deposition rate is low, the nuclei can have the same orientation as the parent quartz crystal, forming the epitaxial waves. In contrast, when the temperature is low and the deposition rate is high, the nuclei can be misoriented with respect to the parent quartz crystal, leading to spherulitic fibers. Sometimes, the competition between the epitaxial growth and the spherulitic growth can be close, as in the case of the epitaxial wave structures, and local relaxation with a gradual change of crystallographic orientation is observed. In addition, we show that by using a TiO_2_ buffer layer, which separates the thin films and the substrates, the α–β phase transition can be avoided, improving the thermal stability of GeO_2_ with respect to that of the SiO_2_ films.

## Supporting Information Available

Additional information on AFM of the TiO_2_ layer; in-plane orientation of TiO_2_ by EBSD; height profile along the crystallization direction; EBSD of Dauphine twining; EBSD of GeO_2_ on TiO_2_-buffered Y-cut quartz with a large zoom-in characterization of GeO_2_ films annealed at 730 °C; and magnitude of the lattice rotation along one fiber by EBSD (PDF)
