# Supplementary material for: Thin Films of α-Quartz GeO2 on TiO2-Buffered Quartz Substrates
Source: Cryst Growth Des. 2023 Dec 18;24(1):71–8. doi: 10.1021/acs.cgd.3c00476 (PMC10767700; doi:10.1021/acs.cgd.3c00476)
Supplement: Supplementary file 1 — cg3c00476_si_001.pdf [file cg3c00476_si_001.pdf]

Supplementary Information of "Thin films of α-quartz GeO2 on TiO2-buffered quartz substrates"

Silang Zhou[1], Kit de Hond[2] , Jordi Antoja-Lleonart[1] , Václav OcelÍk[1] , Gertjan Koster[2] , Guus Rijnders[2] and Beatriz Noheda[1*]

1.Zernike Institute for Advanced Materials, University of Groningen, Nijenborgh 4, 9747 AG, Gronigen, The Netherlands

2. MESA+ Institute for Nanotechnology, University of Twente, PO Box 217, 7522 NH Enschede, The Netherlands

E-mail: b.noheda@rug.nl

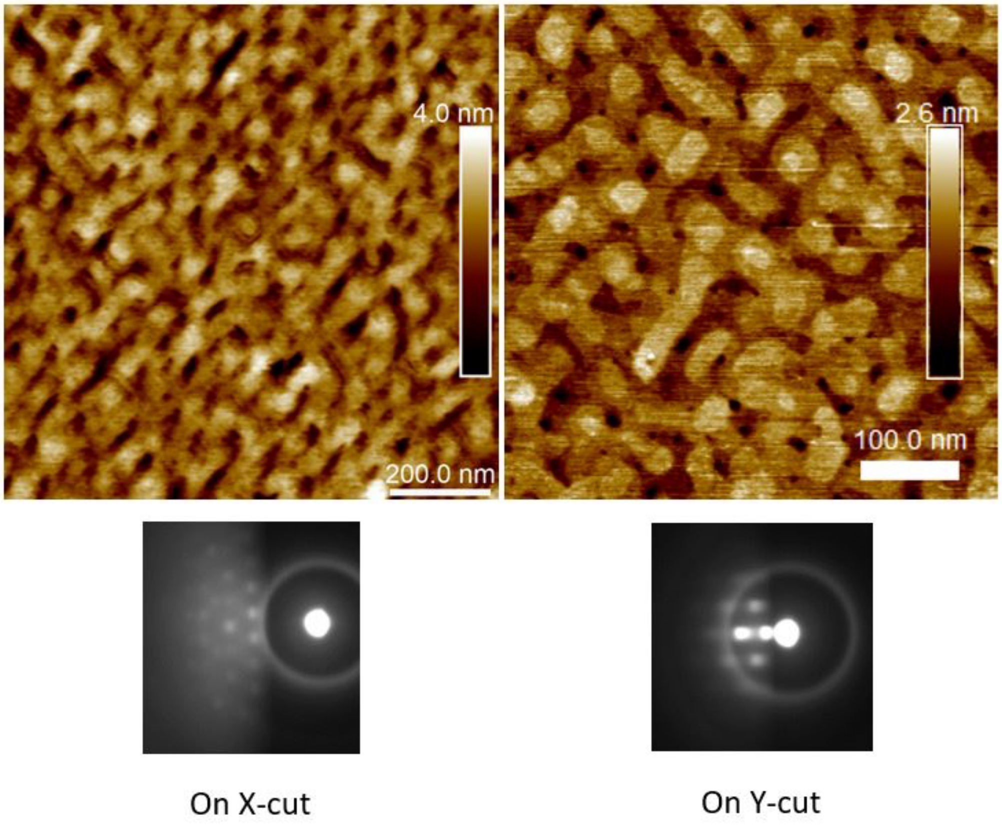

Figure S 1. AFM images and RHEED pattern of the TiO_2 layer on X-cut (left) and Y-cut (right) quartz substrates.

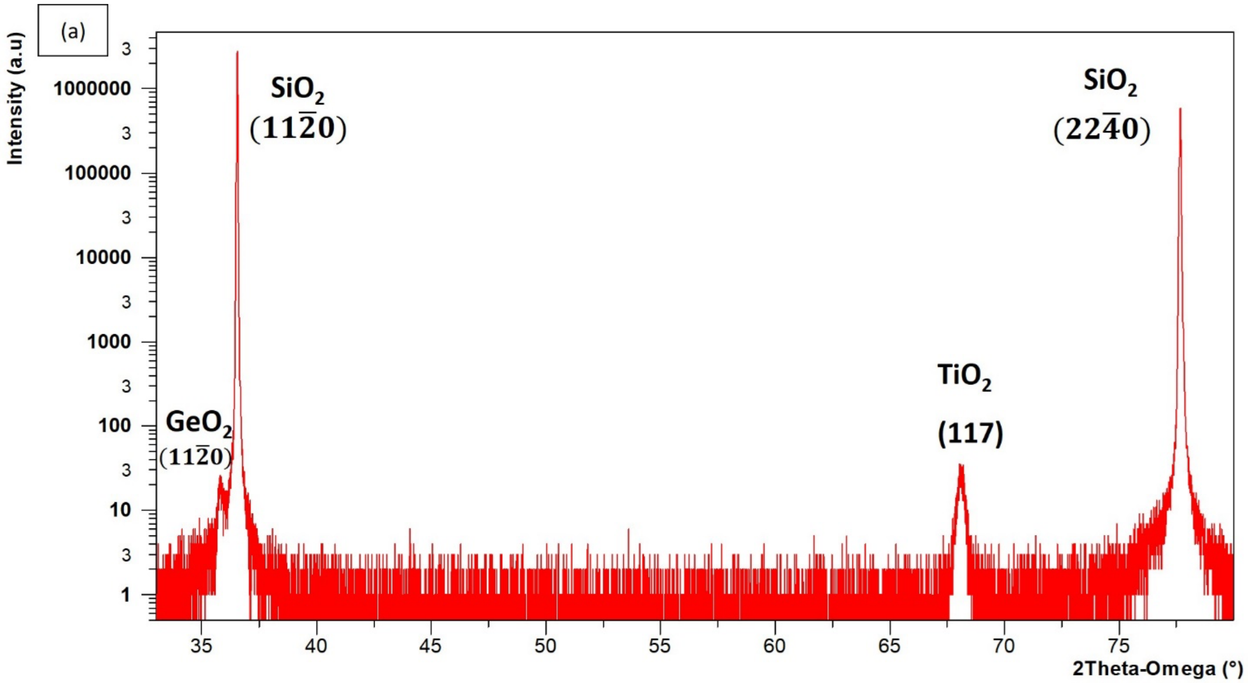

(a)
Intensity (a.u)
1000000
100000
10000
1000
100
10
1
3
SiO2 (11$\bar{2}$0)
SiO2 (22$\bar{4}$0)
GeO2 (11$\bar{2}$0)
TiO2 (117)
35
40
45
50
55
60
65
70
75
2Theta-Omega (°)

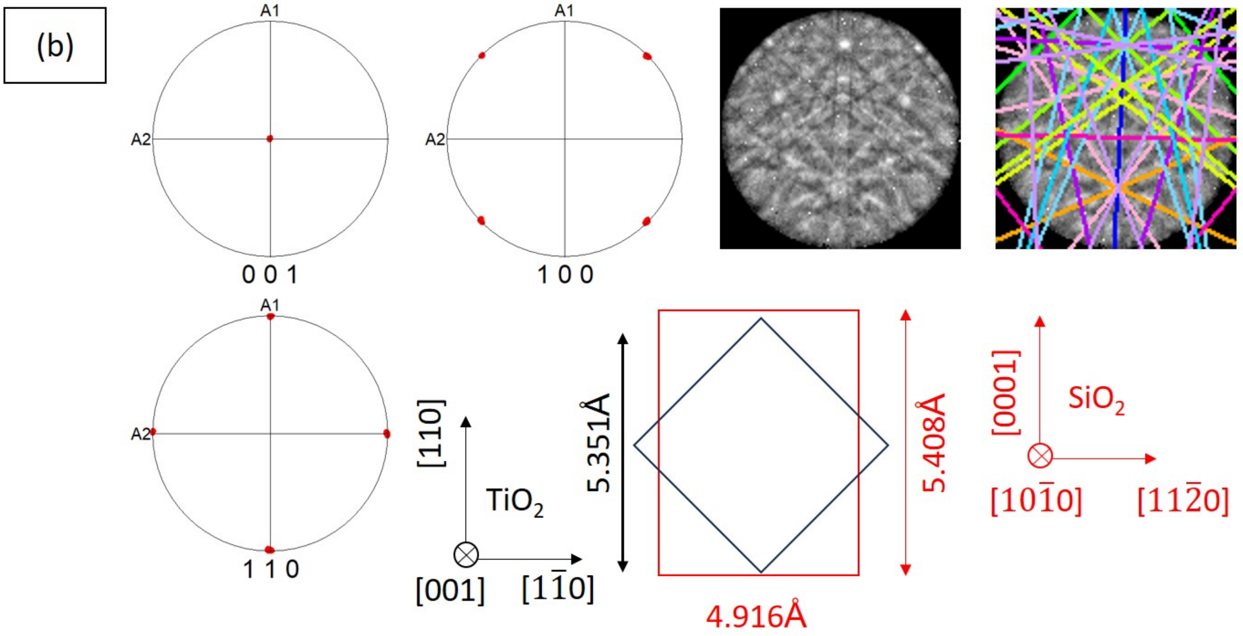

(b)
A1
A2
0 0 1
1 0 0
1 1 0
[110]
TiO2
[001]
[1$\bar{1}$0]
5.351Å
5.408Å
4.916Å
[0001]
SiO2
[10$\bar{1}$0]
[11$\bar{2}$0]

(c)

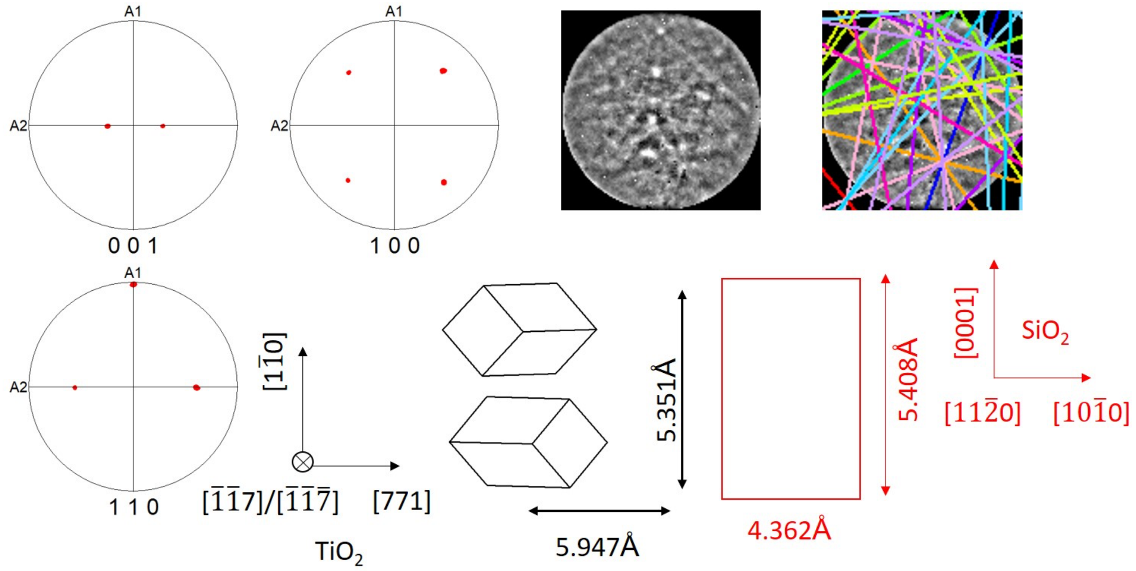

Figure S 2.(a) 2ϑ-ω scan of films grown on X-cut quartz substrates, showing TiO2 orients with (117) out-of-plane. (b), (c): In-plane orientation of TiO_2 on Y-cut substrate and X-cut substrates, respectively.

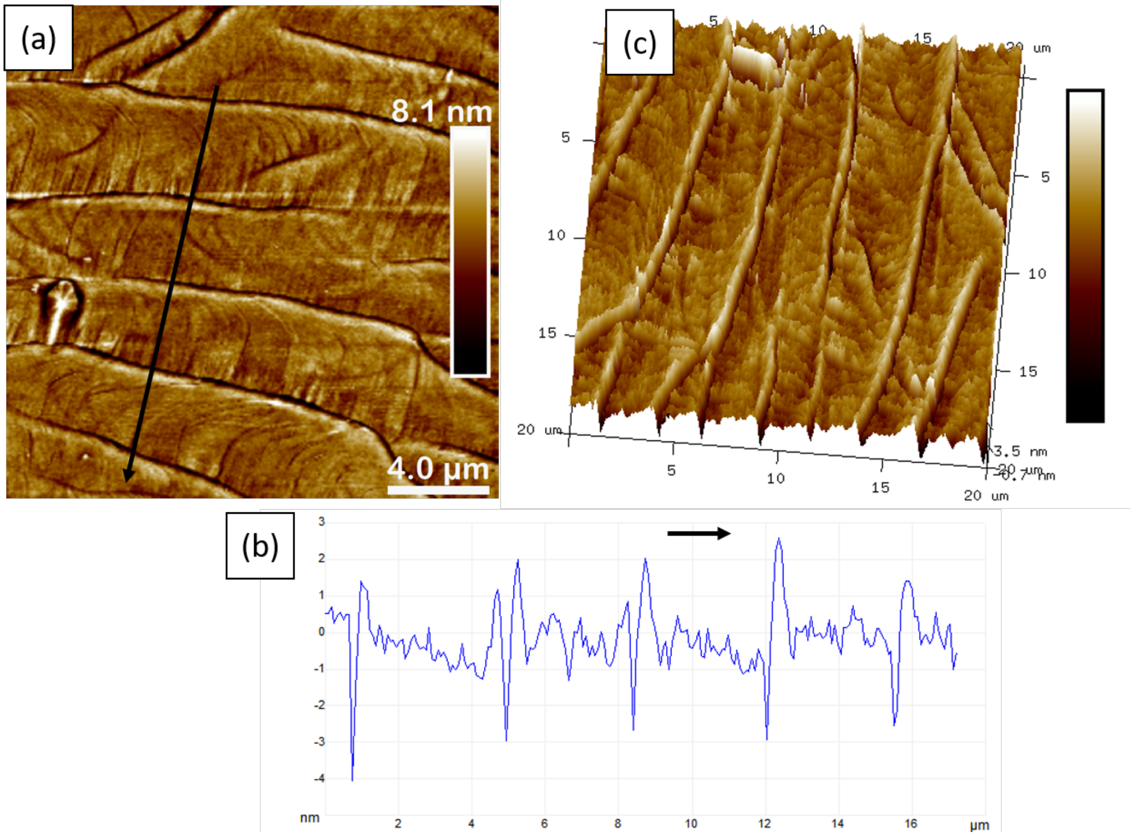

Figure S 3. (a) the same AFM image in Figure3 in the main text. (b) Height profile along the black line in (a) showing semi-periodic height changes. (c) 3-dimentional view of (a).

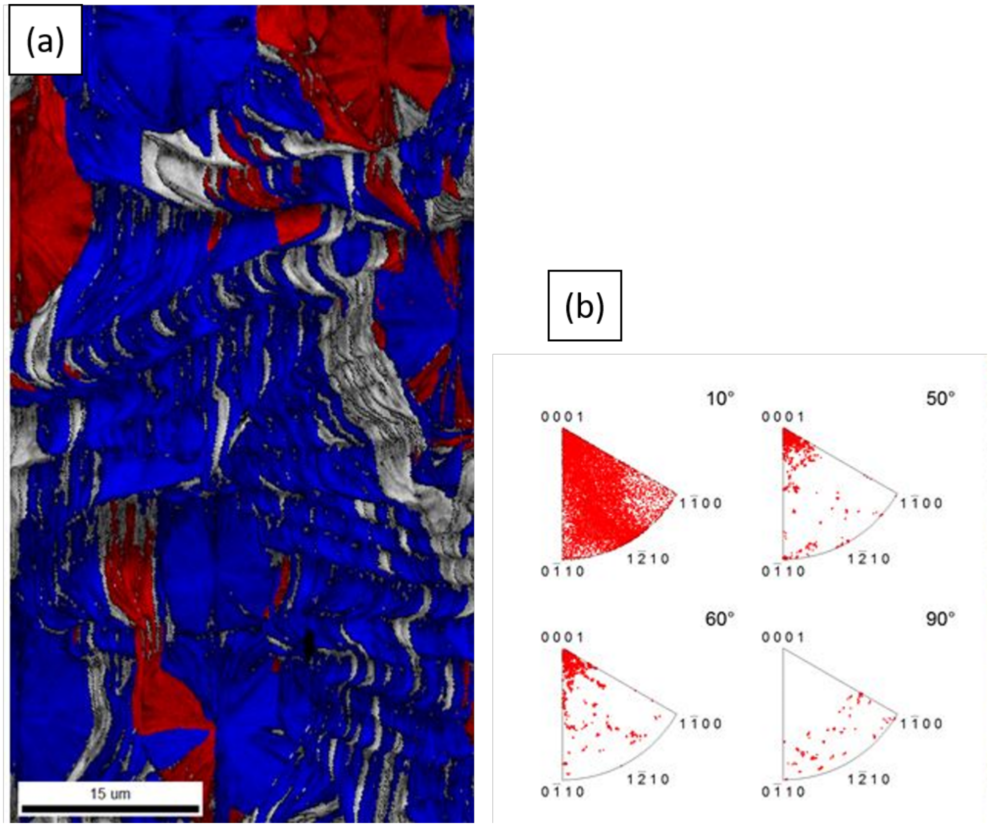

Figure S 4.(a) EBSD map of twinning of Figure 4 in the main text, where blue and red crystals are Dauphine twins with each other. (b) shows the distribution of rotation axis directions for different misorientation angles between the neighbor grains. It is clear that for misorientation angle about 50-60 degree, the rotation axis is close to [0001], which corresponds with the twining axis of Dauphine twins.

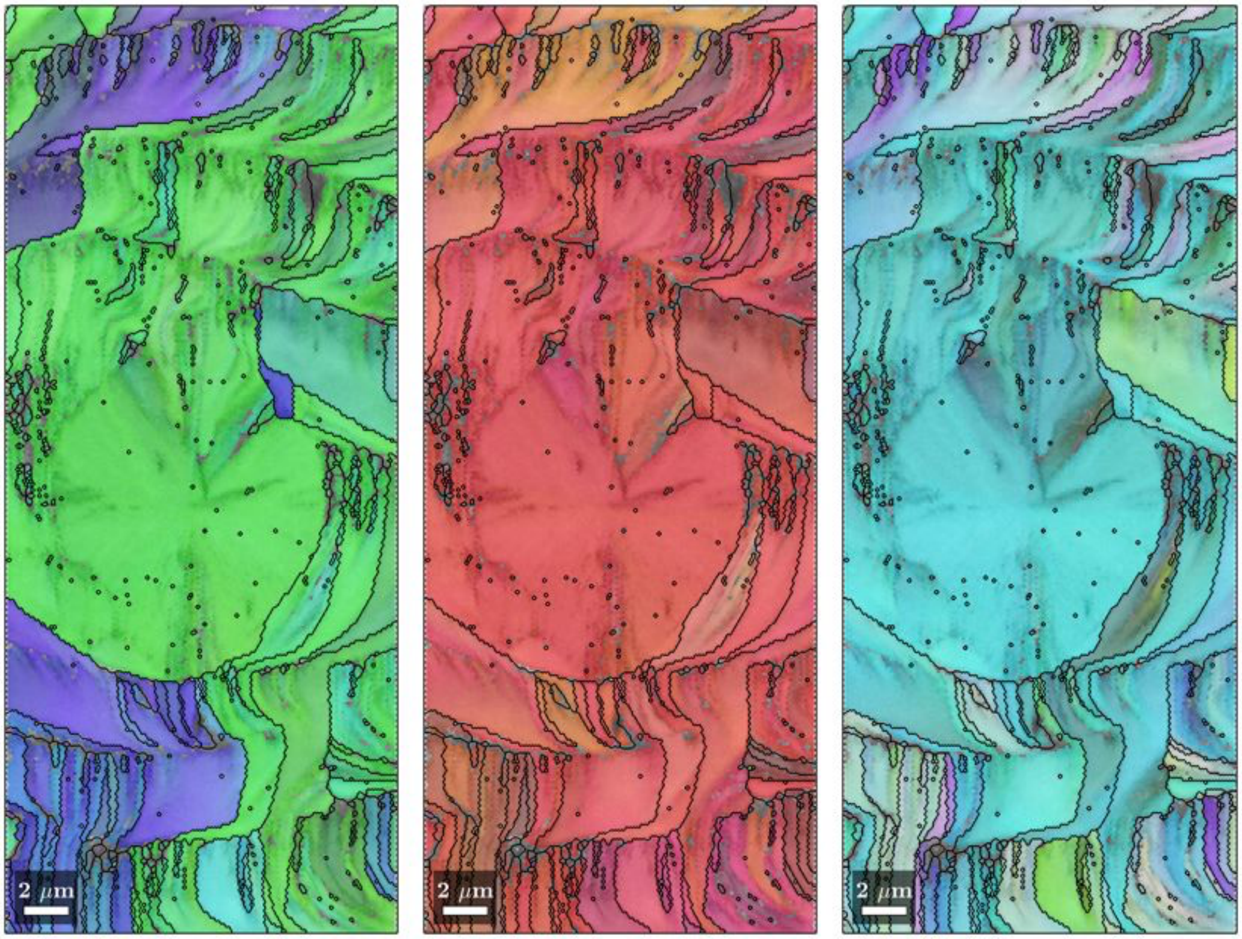

Figure S 5. Overlay of EBSD IQ map and IPF maps viewed from [100], [010], [001] directions, respectively. It is clear that at in some of the wave-like structures, during the growth, the orientation of the crystal gradually differs with neighboring area and finally becomes a new grain. The black lines are the grain boundaries with misorientation angle larger than 3◦. The same color legend is used as in Fig. 4 in main text.

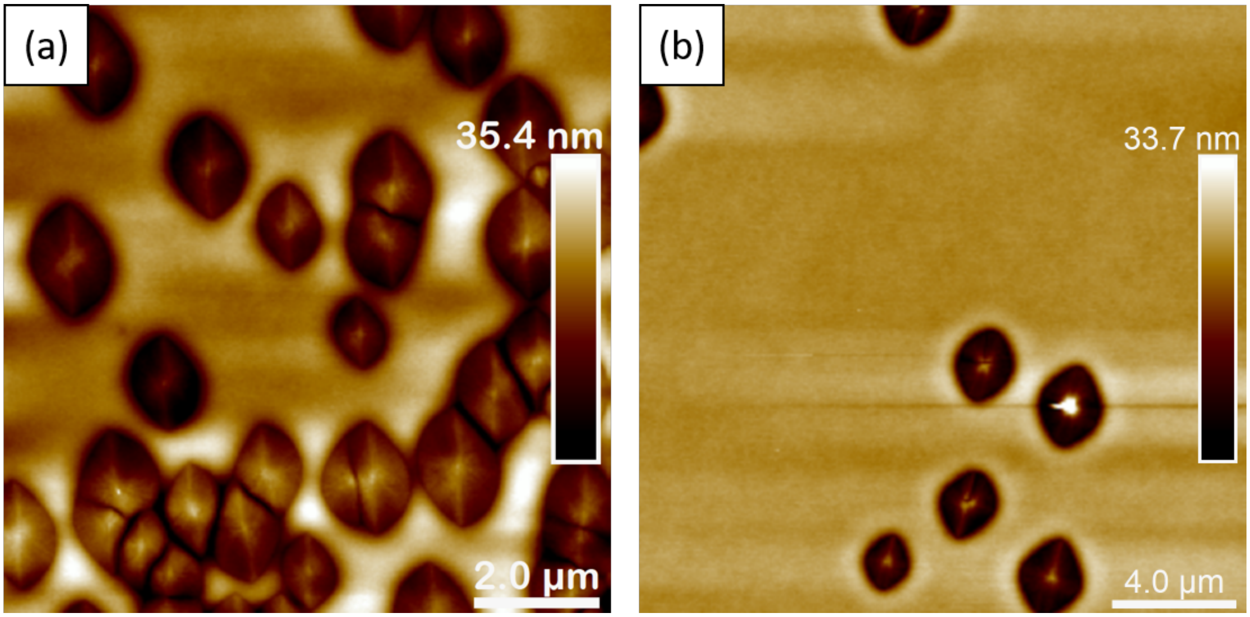

Figure S 6. AFM images of leaf crystals on (a) TiO_2-bufferd X-cut substrates, (b) TiO_2-bufferd Y-cut substrates. In (a), all the (separate) leaf crystals are not symmetric with respect to their long axis while all the leaf crystals in (b) are symmetric with respect to their long axis.

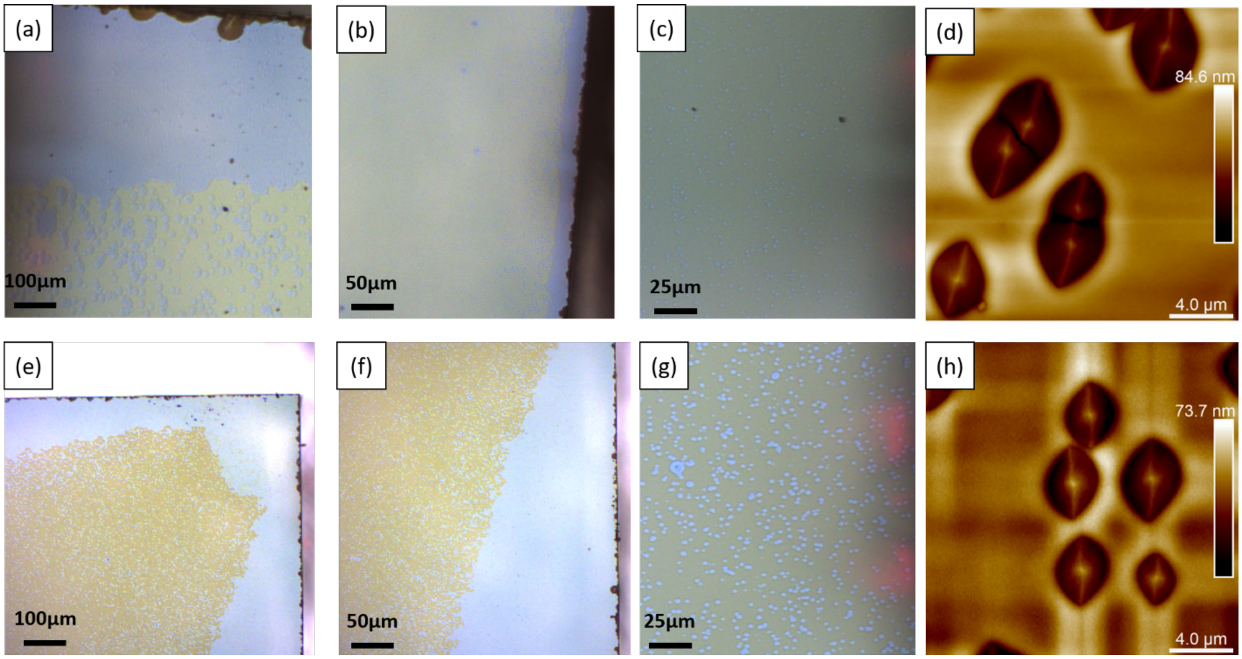

Figure S 7. Thin films of GeO_2 on TiO_2-bufferd X-cut substrates annealed at 730$℃$*°for: (a)-(d), 30 minutes and (e)-(h), 2 hours. (a), (b), (e) and (f) are from the edges of the samples. (a) shows clear nucleation gradient at the sample edge. (e) and (f) show the spherulitic crystallization front with an arc shape advance to the inside of the sample during the annealing. (c) and (g) are the*

center of the sample. It can be observed the density of the leaf crystals in (g) are higher than in (c) due to longer annealing time. (d) and (h) show with longer annealing time, the size of the leaf crystals didn't change much.

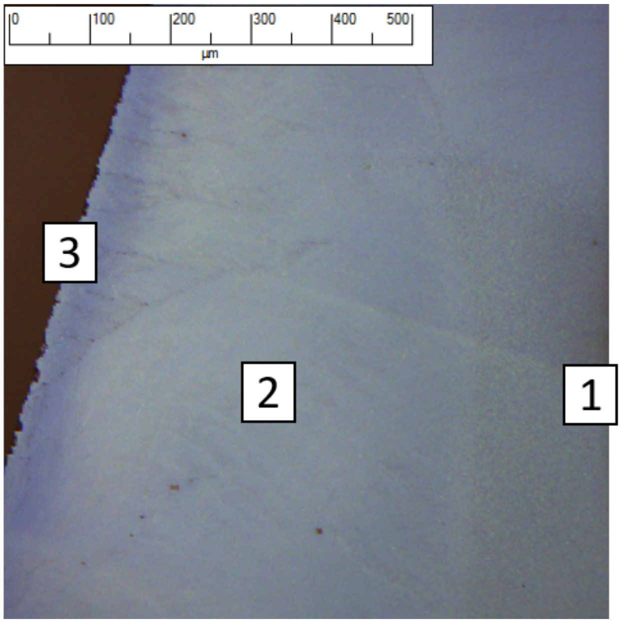

Figure S 8. Optical image of the edge of the sample in Figure5 in the main text showing clear contrast for different areas. The growth front of area 2 is an arc, suggesting spherulitic growth.

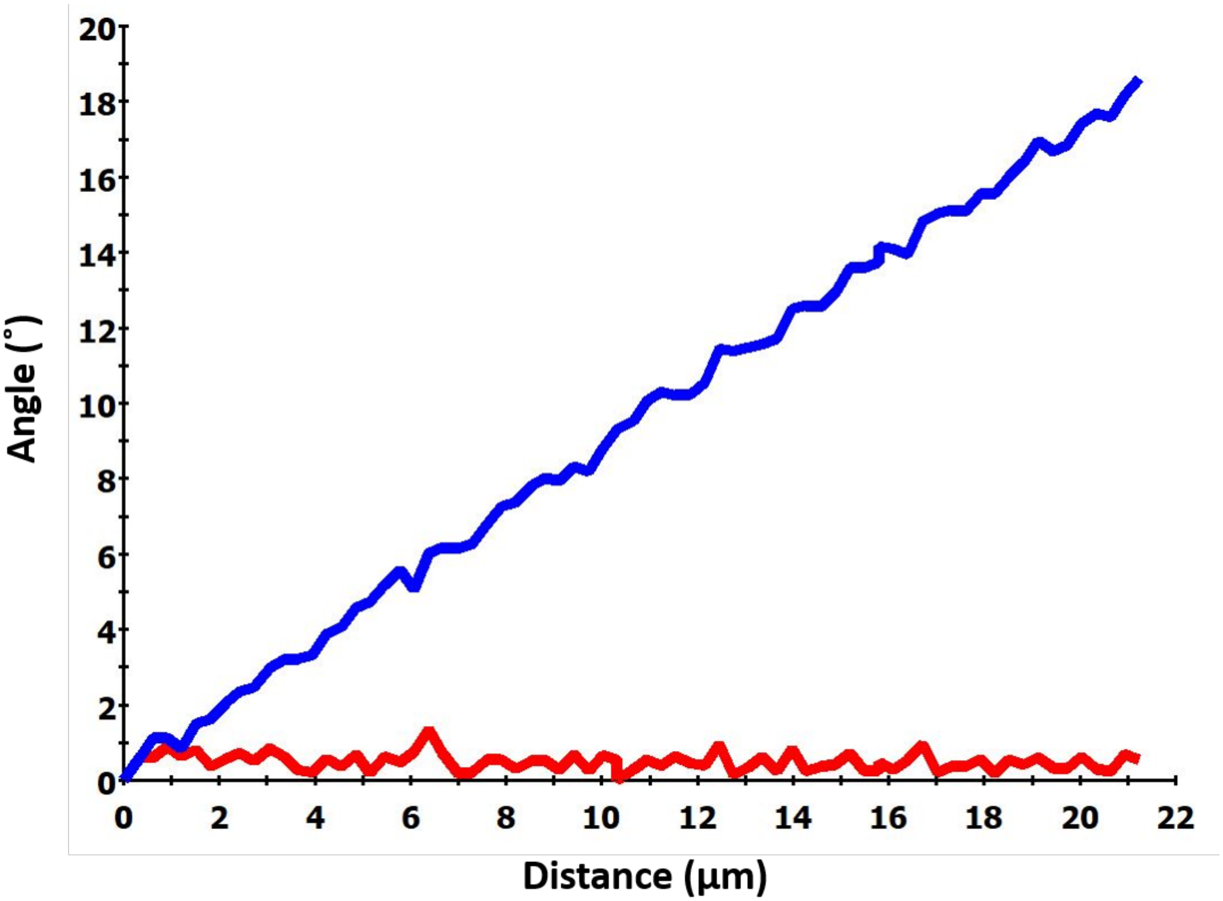

Figure S 7. An example of the lattice rotation determined by EBSD along one fiber where the misorientation angle increases linearly with the distance. Blue line shows the misorientation angle between the point and the origin of measurement, while the red line shows the misorientation angle between neigbouring points.
